# Efficacy of resin-based and glass-ionomer sealants in retention, fissure penetration, and occlusal caries inhibition: an in vitro study

**DOI:** 10.1007/s40368-025-01157-9

**Published:** 2026-03-27

**Authors:** S. H. C. Yeh, M. M. A. Abdalla, P. P. Y. Lam, C. K. Y. Yiu

**Affiliations:** Faculty of Dentistry, University of Hong Kong, Hong Kong, China

**Keywords:** Fissure sealant, Penetration, Retention, Dental caries, Mineral density

## Abstract

**Purpose:**

To compare the efficacy of resin-based sealants (RBS) and glass-ionomer sealants (GIS) with varying viscosities on retention, fissure penetration and occlusal caries inhibition under simulated oral conditions.

**Methods:**

Extracted human third molars were randomly assigned to five groups: high-viscosity GIS (Group 1), medium-viscosity GIS (Group 2), low-viscosity GIS (Group 3), RBS (Group 4), and no sealant (Group 5, negative control). Sealant retention was evaluated visually after 37 °C water storage and thermocycling of 10,000 cycles between 5 and 55 °C with a dwell time of 20 s, whilst penetration depth was assessed using scanning electron microscopy (SEM). Mineral density changes (ΔMDV) following pH cycling for 21 days and bacterial challenge with *Streptococcus mutans* for 7 days were quantified using micro-CT scans. Statistical analysis included Kruskal–Wallis tests, Mann–Whitney *U* tests with Bonferroni correction, Chi-square tests, Spearman’s correlation, and multiple linear regression analyses.

**Results:**

All sealants exhibited optimal retention (> 83% fully intact) without significant differences (*p* = 0.494). RBS demonstrated superior penetration (median: 100%), significantly higher than high-viscosity GIS (median: 90.5%, *p* = 0.003). RBS also provided the greatest protection against mineral loss under both pH cycling (IQR: 0.0252) and bacterial challenges (IQR: 0.0349), exhibiting significantly lower ΔMDV compared to majority of the GIS groups and the negative control (*p* < 0.001). No significant correlation was observed between sealant penetration or retention and ΔMDV outcomes.

**Conclusions:**

All sealants showed effective retention and penetration capabilities; however, the RBS provided superior caries inhibition compared to glass-ionomer alternatives. The effectiveness of GIS was comparable amongst varying viscosities.

## Introduction

Dental caries, or tooth decay, is a prevalent disease that predominantly affects children and adolescents across the globe, with considerable detrimental effects on their overall and oral health-related quality of life (OHRQoL) (Uribe et al. 2021; Wen et al. 2022). The repercussions of dental caries are far-reaching, often resulting in hampered growth, reduced weight gain, and an overall decline in health status (Reis et al. 2025; Yilmaz et al. 2025). The occlusal surfaces of molars are especially prone to dental caries due to the morphology of their pits and fissures, which are more conducive to the development of occlusal caries compared to other tooth surfaces (He et al. 2024). These areas exhibit a higher propensity for caries incidence in contrast to other sites of the teeth (He et al. 2024).

Introduced in the 1960s, fissure sealants have become a cornerstone in the preventative strategy against occlusal caries (Kashbour et al. 2020; Lam et al. 2021). They are applied to the occlusal surfaces of molars to seal off deep pits and fissures, thus preventing food and plaque accumulation, and significantly curtailing the likelihood of caries formation (Nunn et al. 2000). Various systematic reviews and guidelines have recommended the use of fissure sealants, especially as a preventive measure against occlusal caries in permanent molars (Kashbour et al. 2020; Lam et al. 2021).

The market offers plenty of sealant materials, amongst which resin-based sealants (RBS) and glass-ionomer sealants (GIS) are the most utilised. Resin-based sealants have been supported by moderate-quality evidence confirming their effectiveness in occlusal caries prevention, an attribute largely credited to their physical retention and enduring clinical performance (Kashbour et al. 2020; Lam et al. 2021).. Studies showed that RBS maintains an over 80% retention rate in permanent molars at 24-month follow-up and in primary molars after 18 months (Kashbour et al. 2020; Lam et al. 2021). The application of RBS is considered to be more cost-effective compared to no sealant application, especially for high-caries-risk children who are more likely to require dental restorations. School-based RBS programmes have been recommended due to their cost efficiency and effectiveness comparable to those offered in private practises (Lam et al. 2021). However, RBS application is recognised as more technique-sensitive, necessitating a higher level of cooperation from patients. Proper moisture control and acid etching are essential prerequisites for achieving satisfactory RBS retention (Muller-Bolla et al. 2006).

Conversely, GIS exhibits a notably lower retention rate than RBS, with a general retention rate of around 20% for all types of GIS in both permanent and primary molars at the 24-month mark (Lam et al. 2021).Nevertheless, the caries-preventive efficacy of GIS has been found to be on par with that of RBS (Lam et al. 2021). GIS have several operational advantages, including resistance to moisture and a simplified application process that obviates the need for a bonding agent or acid etching. This simplicity translates into reduced complexity and duration of the procedure. The hydrophilic nature of GIS confers it with superior moisture resistance through chemical adhesion to tooth surfaces, unlike RBS, which requires thorough isolation from salivary contamination (Mickenautsch and Yengopal 2016).

In light of the extensive research on dental sealants under various conditions, such as thermocycling, pH cycling, and bacterial exposure, there remains a deficiency in integrative studies that mimic the conditions experienced in the oral cavity and concurrently assess these factors within a unified research framework. Our study sought to address this knowledge gap by conducting a thorough analysis that amalgamated three primary evaluations, thereby dissecting the characteristics of RBS and GIS in a comprehensive manner. Under controlled conditions, direct and quantitative assessments of sealant retention and adhesion were conducted. A standardised and precise approach was employed to evaluate sealant penetration—considering factors such as viscosity and fissure shape—which may be difficult to assess clinically. Additionally, the effects of occlusal caries inhibition were measured under these standardised conditions.

This holistic investigation was designed to unravel the intricate interplay between these variables and their collective influence on the performance of dental sealants. The study aimed to investigate and compare the sealant penetration ability, retention, and occlusal caries inhibition of RBS and GIS of different viscosities. The objectives of the study were to compare (i) the sealant retention and fissure penetration between RBS and GIS of varying viscosities under thermocycling, (ii) the change in mineral density under pH cycling, and (iii) the change in mineral density under bacterial testing of RBS and GIS of different viscosities. The null hypotheses tested were that (i) there is no significant difference in sealant retention between RBS and GIS of varying viscosities; (ii) there is no significant difference in fissure penetration, between RBS and GIS of varying viscosities; and (iii) there is no significant difference in the change in mineral density under pH cycling and bacterial testing of RBS and GIS of different viscosities.

## Materials and methods

### Experimental study design

**Fig. 1:**
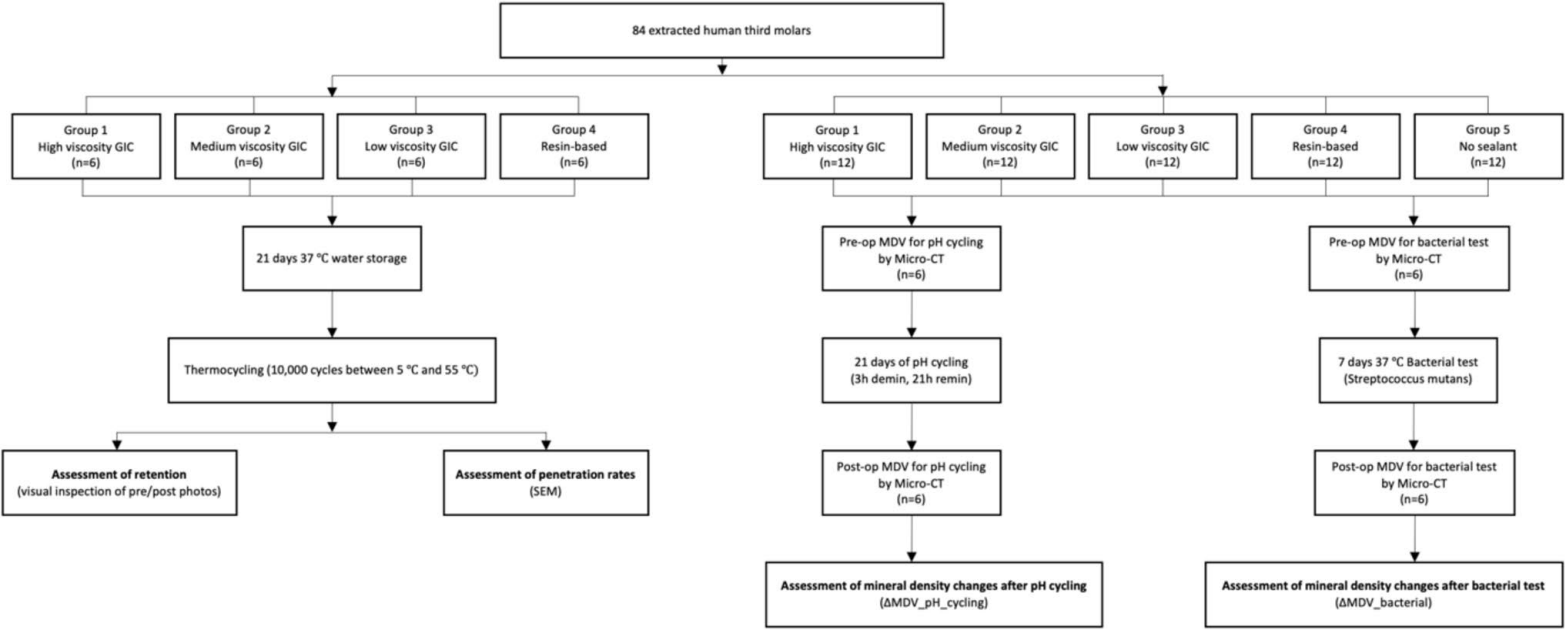
Flowchart of the study

The experimental study design and ethical considerations were reviewed and approved by the Institutional Review Board of The University of Hong Kong–Hospital Authority Hong Kong West Cluster (Reference number: UW 20–293). A schematic overview of the study design is illustrated in Fig. 1, outlining the key stages of the experimental process. This study involved an in vitro assessment of tooth specimens, which underwent artificial ageing through thermocycling and the cariogenic challenges via pH cycling or bacterial test. Subsequent investigations focussed on fissure penetration ability, sealant retention, and caries inhibition efficacy.

Sample size calculation was conducted using G*Power 3.1 (Franz Faul, Germany). Each molar could be further sectioned into three specimens for testing. Based on parameters derived from the previous study (Kramer et al. 2018), with a statistical power of 0.95 and a significance level of 0.05, the minimum required sample size was determined to be 18 specimens per group across the five study groups, derived from six molars. When investigating fissure penetration and sealant retention, 24 molars (i.e., 6 molars × 3 specimens per tooth × 4 study groups = 72 specimens) were used for thermocycling as negative control was not required. However, for pH cycling and bacterial challenge, 30 molars (i.e., 6 molars × 3 specimen per tooth × 5 study groups = 90 specimens) were needed for each experiment to evaluate the caries-inhibitory potential of sealants compared to no sealants. Hence, a total of 84 permanent molars were used, providing 252 specimens for the investigations.

### Specimen preparation

Freshly extracted, caries-free, intact, unrestored human third molars were collected for therapeutic reasons from patients after obtaining informed consent. To assess the immediate effects of the sealant materials and reduce influence of confounding factors, the specimens used in the study were not aged. Following surface debridement with ultrasonic scaler, the teeth were carefully inspected under a stereomicroscope at 8 × magnification (Carl Zeiss Stereo 475,002, Oberkochen, Germany). Any teeth presenting with enamel defects, such as fluorosis, hypomineralisation, or hypoplasia, were excluded from the study. The teeth that met the inclusion criteria were then immersed in 0.5% thymol solution maintained at 37 °C until use.

The roots of the selected teeth were removed by sectioning at the cementoenamel junction using a custom-made saw microtome *(SYD Miki Pulley, Kawasaki, Japan)* with a 150-μm-thick blade. Except for the occlusal surface, acid-resistant nail varnish was sealed to all the other surfaces (Revlon, New York, NY, USA). The prepared teeth were then randomly divided into 5 groups (*n* = 6), each designated to receive a different type of sealant material on the occlusal surface fissures, applied according to the respective manufacturer's instructions (Table 1).

**Table 1:** Sealants used and manufacturer' instructions

Group	Sealant (manufacturer)	Active component	Pretreatment conditioning	Procedure
G1	Ketac™ Molar Easymix (3 M ESPE, MN, USA)	High-viscosity glass-ionomer	Polyacrylic acid solution (Ketac™ Conditioner; 3 M ESPE, MN, USA)	1. Apply polyacrylic acid solution to further clean the occlusal surface (10 s) 2. Rinse thoroughly and dry the tooth surface 3. Mix powder and liquid in a standardised ratio (1 spoonful of powder to one drop of liquid) 4. Apply with finger press technique with a gloved finger and wipe away the excess 5. Apply Vaseline immediately after placement
G2	GC Fuji VII (GC, Tokyo, Japan)	Medium-viscosity glass-ionomer	Polyacrylic acid solution (GC Cavity Conditioner, GC, Tokyo, Japan)	1. Apply GC Cavity Conditioner to further clean the occlusal surface (10 s) 2. Rinse and slightly dry the tooth 3. Extrude the mixture onto the tooth surface, then spread a thin film of GC Fuji VII over occlusal surface, including pits and fissures with microbrush 4. Apply Vaseline immediately after placement
G3	GC Fuji TRIAGE (GC, Tokyo, Japan)	Low-viscosity glass-ionomer	Polyacrylic acid solution (GC Cavity Conditioner, GC, Tokyo, Japan)	1. Apply GC Cavity Conditioner to further clean the occlusal surface (10 s) 2. Rinse and slightly dry the tooth 3. Extrude the mixture onto the tooth surface then spread a thin film of GC TRIAGE over occlusal surface, including pits and fissures with microbrush 4. Apply Vaseline immediately after placement
G4	Helioseal® (Ivoclar, Schaan, Liechtenstein)	Bis-GMA (bisphenol A-glycidyl methacrylate) based resin, triethylene glycol dimethacrylate	37% phosphoric acid (Scotchbond™, 3 M ESPE, MN, USA)	1. Apply etching gel and let it react for 60 s 2. Rinse and dry thoroughly and the etched enamel should have a mat white appearance 3. Apply Helioseal directly with microbrush to seal off the fissures 4. Wait for 15 s and cure with curing light (Bluephase®) for 20 s
G5	No sealant	Not applicable	None	None (negative control for pH cycling & bacterial challenge only)

### Water storage and thermocycling

Photographs (settings: F22, exposure time 1/40, ISO 100, EOS 200D; Canon Inc., Tokyo, Japan) were taken after the application of the sealant at baseline as described in Table 1. The specimens were labelled with numbers in random sequences and then stored for 21 days in distilled water at 37 °C and 95% relative humidity in an incubator (Memmert Inc., Schwabach, Germany). Following the storage period, each tooth specimen underwent a thermocycling period of 10,000 cycles between 5 and 55 °C with a dwell time of 20 s each (AlQahtani et al. 2022).

### Sealant retention evaluation

After the thermocycling, the retention of the sealant was first evaluated with the naked eye without any magnification. Referring to the photographs taken at baseline, sealant retention was assessed and classified as "fully intact", "partially intact", or "totally loss" (Li et al. 1981; Mascarenhas et al. 2008).

### Sealant penetration evaluation

Each tooth was further sectioned at two sites in a buccolingual plane using a custom-made water-cooled diamond-impregnated low-speed saw, yielding three sectioned surfaces per specimen for analysis using scanning electron microscopy (SEM) (SU1510, Hitachi, Chiyoda, Japan). The specimens were mounted on aluminium stubs of the SEM, sputter-coated with platinum–palladium *(80% platinum and 20% palladium; IXRF Systems, Inc., Austin, TX, USA)*, and examined using an SEM (Markovic et al. 2011). Sealant penetration was evaluated at 45 × magnification.

**Fig. 2:**
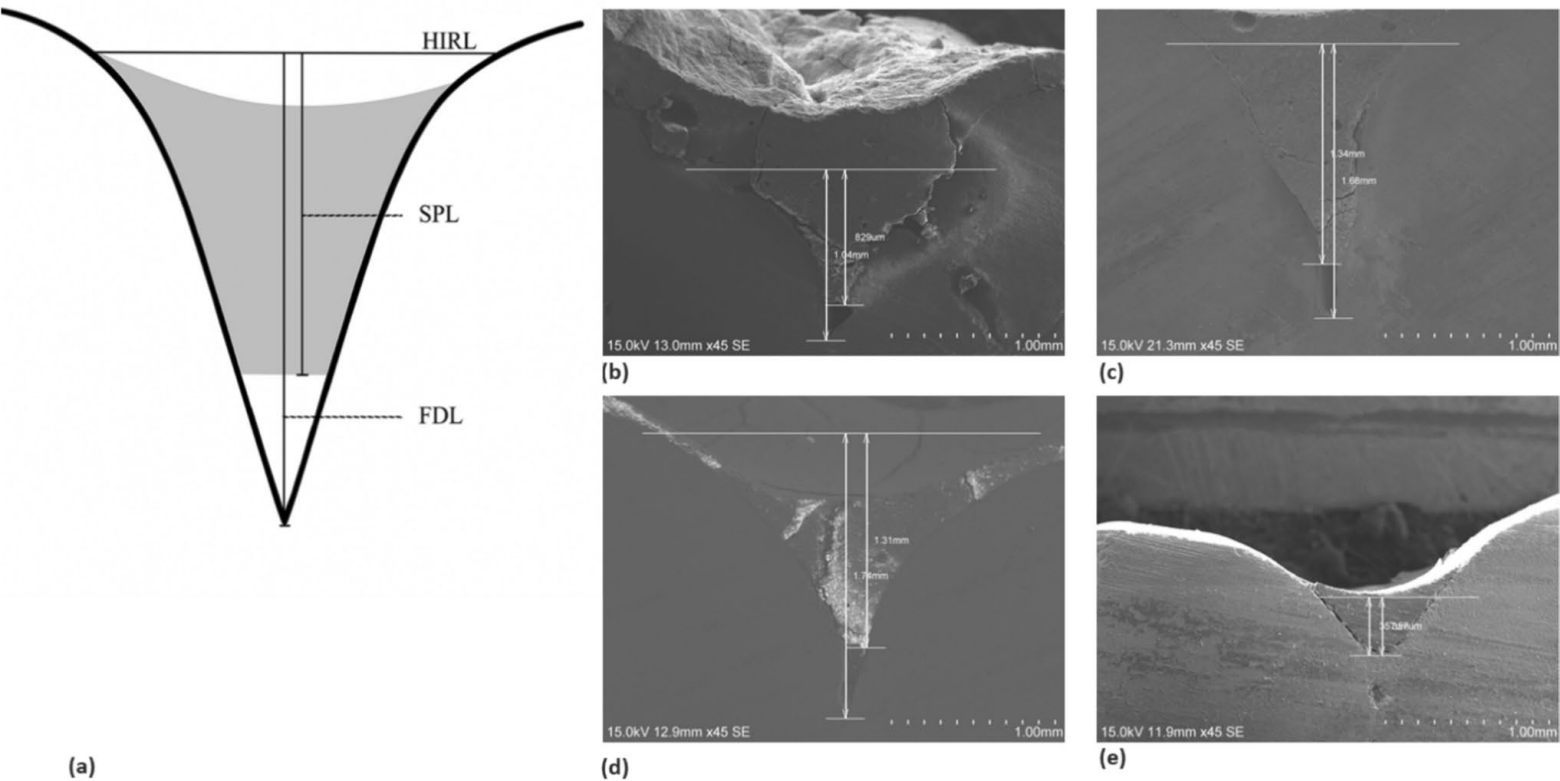
SEM for sealant penetration assessment (**a**) Schematic drawing of horizontal intercuspal reference line (HIRL), fissure depth line (FDL), and sealant penetration line (SPL) measurements; **b** high-viscosity GIS (Ketac Molar), **c** medium-viscosity GIS (Fuji VII), **d** low-viscosity GIS (Fuji TRIAGE), **e** Resin-based sealant (Helioseal)

In each image, a calibrated horizontal line was drawn at the top as a reference line to assess the penetration depth in relation to the fissure depth. Another line, drawn below the buccal and lingual slopes perpendicular to the cusp section, served as a horizontal intercuspal reference line (HIRL). A fissure depth line (FDL) is a line drawn from, and perpendicular to, the HIRL to the bottom of the fissure. Sealant penetration line (SPL) was measured from the lowest point where the sealant is present to the HIRL on the FDL. Due to the uneven sealant surfaces, HIRL was utilised instead of the upper margin to reduce potential bias and standardised the evaluation from different fissure morphologies. Sealant penetration was calculated with the fraction SPL/FDL and expressed as a percentage. The assessment of sealant penetration was performed by one examiner (first author) who received training and calibration from a senior research (third author) with photographs of the past similar studies. Figure 2 shows the schematic drawing of HIRL, FDL, and SPL measurements.

Sealant penetration ability was assessed across the four groups using SEM. Eighteen measurements were recorded for each group and statistical comparisons were performed amongst the groups.

### Mineral density assessment

Micro-computed tomography (Micro-CT) scans (Skyscan 1276 X-Ray Microtomograph, Bruker, Kontich, Belgium) were used to assess the mineral density (MD) of the tooth specimens before and after pH cycling and bacterial test, following standard protocols (Bijle et al. 2018). The pixel size for scanning was set at 14 μm. The X-ray beam was directed parallel to the occlusal surface of the specimen mounted on the computer-controlled turntable. Each specimen was scanned with standardised voltage (90 kV), current (200 μA), integration time (3.63 s), and isotropic resolution (14 μm), rotating at 360° with 1° steps. Hydroxyapatite discs with known mineral densities (0.25 g/cm^3^ and 0.75 g/cm^3^) were used as reference phantoms for calibration. The 3D scanned images were reconstructed with NRecon v. 1.7.0.4 (Skyscan 1276 X-Ray Microtomograph, Bruker), using standardised variables (Smoothing: 1; ring artefact correction: 2; dynamic range: 0–0.25) at all scanning levels. Data Viewer v. 1.4.4.0 (*Skyscan 1276 X-Ray Microtomograph, Bruker*) was used to align the preoperative and postoperative reconstructed images in a uniform orientation. The sagittal section was selected for the assessment of mineral density.

The locations corresponding to one-third, one-half, and three-fourths of each specimen were identified and marked for analysis. At each of these points, a cylindrical region of interest (ROI) with a volume of approximately 7.2 mm^3^ (calculated using an area of 0.6 mm^2^ and a height of 12 mm) was established. The mean mineral density value (MDV) within each ROI was determined from micro-CT scans. Consequently, three mean MDV measurements were recorded for each specimen. Preoperative and postoperative micro-CT images were carefully aligned and calibrated to maintain consistent orientation, ensuring that MDV measurements pre- and post-operation were obtained from precisely matched anatomical locations.

### Demineralising and remineralising solutions

Buffered remineralising and demineralising solutions were prepared using analytical grade chemicals and deionised water (Bijle et al. 2018; Gopalakrishnan et al. 2017). The demineralising solution contained 2.2 mM calcium chloride dihydrate (CaCl_2_ × 2H_2_O), 1.35 mM potassium dihydrogen phosphate (KH_2_PO_4_), and 0.05 M acetic acid. The pH was adjusted to 4.4 with 5 M KOH. The remineralising solution consisted of 1.5 mM CaCl_2_ × 2H_2_O, 0.9 mM sodium dihydrogen phosphate (NaH_2_PO_4_), and 0.15 M potassium chloride (KCl). The pH was adjusted to a neutral pH of 7.0 with KOH. This created a state of supersaturation of apatite minerals (Sigma Aldrich, St. Louis, USA), which simulated the conditions found in saliva.

### pH cycling

Following the standardised protocol from previous studies (Yu et al. 2018a, b), a 21-day pH cycling system was used to perform pH cycling on all specimens using an orbital shaker (Labnet, Woodbridge, NJ, USA), operated at 80-rpm at room temperature (Yu et al. 2018a, b). Each daily cycle involved one demineralisation phase lasting for 3 h, alternating with one remineralisation phase lasting for 21 h (Arnaud et al. 2010; Ogawa et al. 2022). To verify the ionic concentrations, a pH metre was used to monitor the pH. The pH was adjusted and maintained at 4 for the demineralization phase and at 7 for the remineralization phase. The solutions were also replaced every 48 h to maintain ionic concentrations. Each specimen was immersed in 5 mL of the treatment solution during each phase individually. Between each phase, deionised water and dry fibreless laboratory-used napkins (Kimwipes™ Ex-L, Kimberly-Clark Professional, *Irving, TX,* USA) were used to rinse and dry the specimens. Following the pH cycling, the specimens were examined again with micro-CT scans *(Skyscan 1276 X-Ray Microtomograph, Bruker)*, adhering to the previously mentioned protocol. The degree of demineralisation at the sealant–enamel interface was analysed.

The mineral density changes (ΔMDV) for each specimen were determined by calculating the difference between the preoperative (ΔMDV_pre) and the postoperative (ΔMDV_post) mineral density values which were measured in g/cm^3^. The formula used for this calculation was: ΔMDV=MDV pre -MDV post. A positive ΔMDV indicates a loss of mineral density, whilst a negative ΔMDV means an increase in mineral density after the tests. The ΔMDV were then used for statistical analysis.

### Bacterial test

Prior to the bacterial challenge, all specimens underwent sterilisation in an autoclave. *Streptococcus mutans* (*S. mutans*) American Type Culture Collection (ATCC) 35,668 was first cultivated on blood agar plates anaerobically for 2 days at 37 °C. One colony was selected to be cultivated anaerobically in brain heart infusion (BHI) broth. The bacterial cell pellets were then collected and transferred to BHI *(Oxoid Limited, Basingstoke, Hampshire, United Kingdom)* with 5% sucrose. After adjusting the bacterial concentration to McFarland 2 (6 × 10^8^ cells/mL), 1.5 mL of the bacterial culture, along with one tooth specimen, was transferred to a well of a 24-well plate. Then, the plates were incubated anaerobically for 7 days at 37 °C and relative humidity 70% (Yu et al. 2018a, b).

### Occlusal caries inhibition

The biofilm on the tooth specimens was removed with ultrasonic cleansing and the specimens were submitted to Micro-CT for assessment of mineral density, following the previously mentioned protocol. The (ΔMDV) was calculated as mentioned above for statistical analysis.

### Statistical analysis

The data were exported to and analysed using SPSS v. 28 (IBM SPSS® Statistics Inc, *Chicago, IL,* USA), after being organised in MS Office Excel 2016 (Microsoft Office Professional Plus 2016, Microsoft, *Redmond, WA,* USA). The Shapiro–Wilk test and Levene test were carried out to assess the normality and equality of variances, respectively. The Kruskal–Wallis test, followed by Mann–Whitney U tests with Bonferroni correction (α = 0.0083 for the thermocycling test, α = 0.005 for the pH cycling and bacterial tests), was conducted for pairwise comparisons in non-parametric data analysis. Chi-square tests were used for sealant retention analysis. When evaluating the interactions between sealant retention, sealant penetration ability, and mineral density changes under pH cycling and bacterial tests, Mann–Whitney *U* test, Spearman’s rank correlation, and multiple linear regression analyses were conducted. The statistically significant level was set at 0.05.

## Results

### Sealant retention by visual inspection

**Fig. 3:**
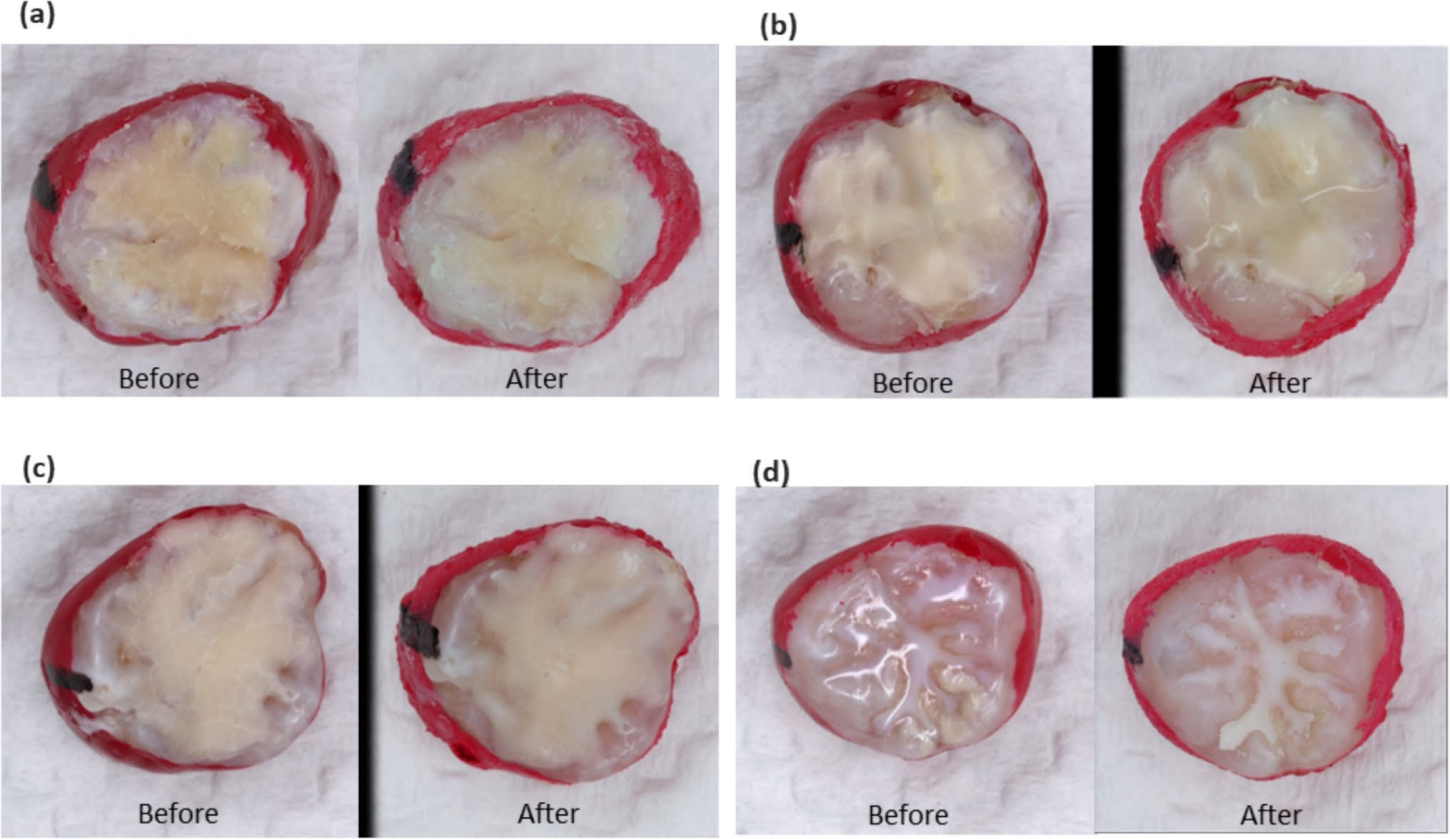
Visual inspection for sealant retention. **a** Group 1: high-viscosity GI sealant (Ketac Molar). **b** Group 2: medium-viscosity GI sealant (FujiVII). **c** Group 3: low-viscosity GI sealant (Fuji TRIAGE). **d** Group 4: resin-based sealant (Helioseal)

Sealant retention was assessed across the four groups by visual inspection after 21 days of water storage and 10,000 cycles of thermocycling (Fig. 3). Of the 24 specimens, 20 sealants were fully intact, 4 were partially intact, and none exhibited total loss. RBS (Group 4) showed the highest retention rate, with all six specimens fully intact, whilst low-viscosity glass-ionomer (Group 3) had the lowest, with four specimens fully intact and two partially intact. High- and medium-viscosity GIS (Groups 1 and 2) each had five fully intact specimens and one partially intact. Chi-square tests indicate that there was no statistically significant difference amongst the groups (p = 0.494).

### Sealant penetration ability by SEM

**Fig. 4:**
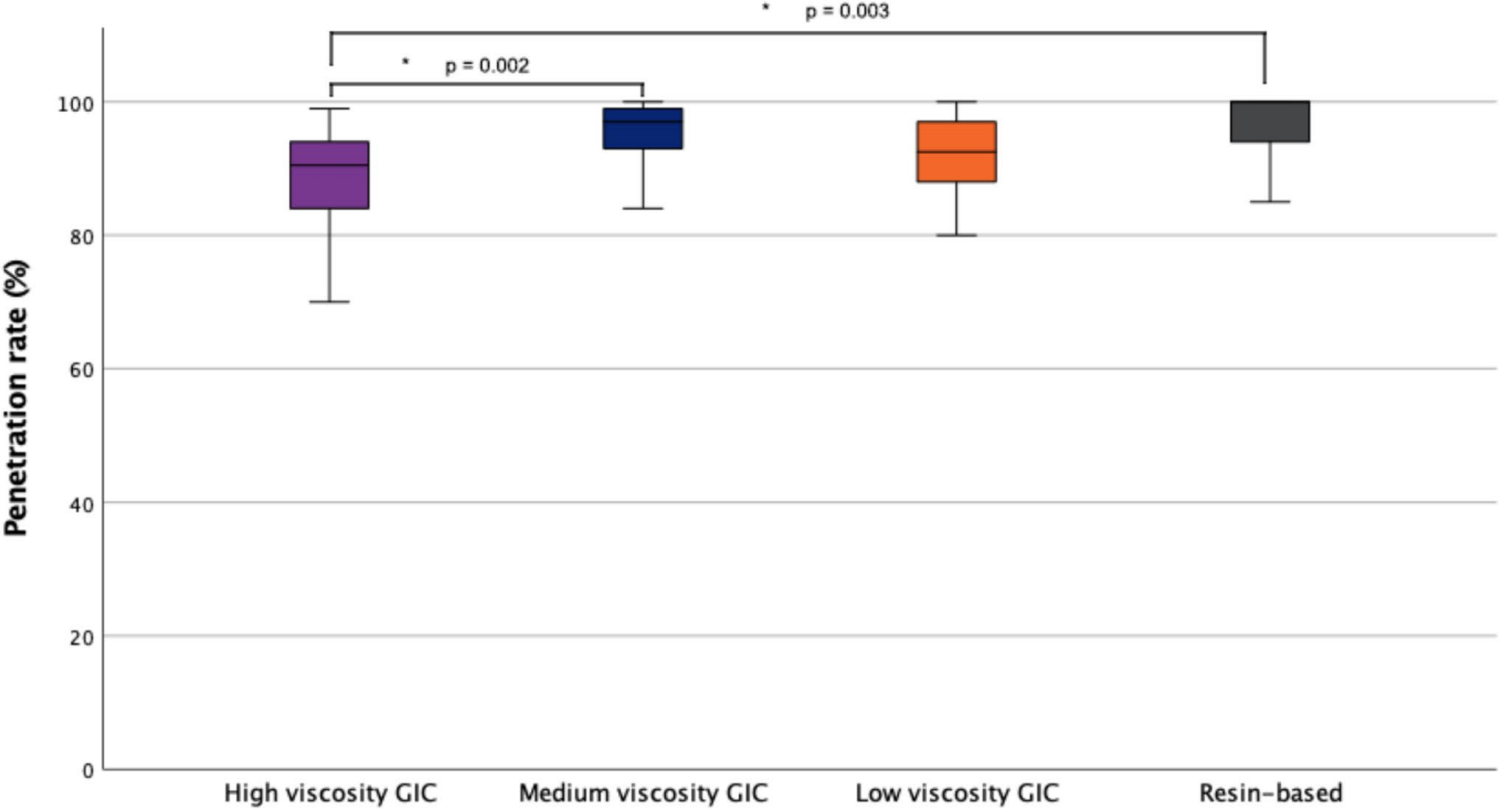
The percentage of sealant penetration rates of the four groups. The asterisk (*) indicates significant differences amongst the groups

Table 2 shows the minimum, maximum, median, interquartile range (IQR) values, and p value of fissure depth, sealant depth, and penetration rates from each group with the pairwise comparisons of penetration rates using Mann–Whitney U tests. The box plot from Fig. 4 shows the penetration rates amongst the groups. Significant differences in fissure depth and sealant depth were observed amongst the groups (p < 0.001), indicating baseline anatomical variability. These variables were not primary outcomes but may have contributed to the differences observed in the penetration rate. Significant differences were observed in the penetration rate between the high-viscosity glass-ionomer (Group 1) and medium-viscosity glass-ionomer (Group 2) (p = 0.002), as well as between the high-viscosity glass-ionomer (Group 1) and RBS (Group 4) (p = 0.003). No significant differences were detected in other pairwise comparisons. The ranking of sealant penetration percentage, based on median values ± interquartile range (%), indicated that the RBS (Group 4) achieved the highest penetration (100 ± 8%), followed by the medium-viscosity GIS (Group 2) (97 ± 6%), the low-viscosity GIS (Group 3) (92.5 ± 10%), and, finally, the high-viscosity GIS (Group 1) with the lowest penetration (90.5 ± 11%).

**Table 2:** Fissure depth, sealant depth, and penetration rate amongst the groups

Variable	Group	Sealant Type	Sealant brand (manufacturer)	Minimum	Maximum	Median	IQR	Kruskal–Wallis *p *value
Fissure depth (μm)	Group 1	High-viscosity glass-ionomer	Ketac™ Molar Easymix (3 M ESPE, MN, USA)	291	1097	736.5	358	*p* < 0.001
	Group 2	Medium-viscosity glass-ionomer	GC Fuji VII (GC, Tokyo, Japan)	366	1091	1044.5	306	
	Group 3	Low-viscosity glass-ionomer	GC Fuji TRIAGE (GC, Tokyo, Japan)	485	1084	1033.5	208	
	Group 4	Resin-based	Helioseal® (Ivoclar, Schaan, Liechtenstein)	229	1054	564.5	530	
Sealant depth (μm)	Group 1	High-viscosity glass-ionomer	Ketac™ Molar Easymix (3 M ESPE, MN, USA)	243	1033	644	280	*p* < 0.001
	Group 2	Medium-viscosity glass-ionomer	GC Fuji VII (GC, Tokyo, Japan)	366	1054	1002	289	
	Group 3	Low-viscosity glass-ionomer	GC Fuji TRIAGE (GC, Tokyo, Japan)	432	1074	903.5	234	
	Group 4	Resin-based	Helioseal® (Ivoclar, Schaan, Liechtenstein)	229	891	564.5	437	
Penetration rate (%)	Group 1	High-viscosity glass-ionomer	Ketac™ Molar Easymix (3 M ESPE, MN, USA)	70	99	90.5^b^	11	*p* = 0.001
	Group 2	Medium-viscosity glass-ionomer	GC Fuji VII (GC, Tokyo, Japan)	84	100	97^a^	6	
	Group 3	Low-viscosity glass-ionomer	GC Fuji TRIAGE (GC, Tokyo, Japan)	80	100	92.5^ab^	10	
	Group 4	Resin-based	Helioseal® (Ivoclar, Schaan, Liechtenstein)	71	100	100^a^	8	

Fissure depth and sealant depth were recorded as anatomical descriptors; penetration rate was the primary outcome of interest. Superscript letters indicate statistically homogeneous subsets based on Mann–Whitney *U* tests with Bonferroni correction (*p* ≤ 0.0083). Groups sharing a letter are not significantly different.

### Mineral density changes (ΔMDV) after pH cycling

**Fig. 5:**
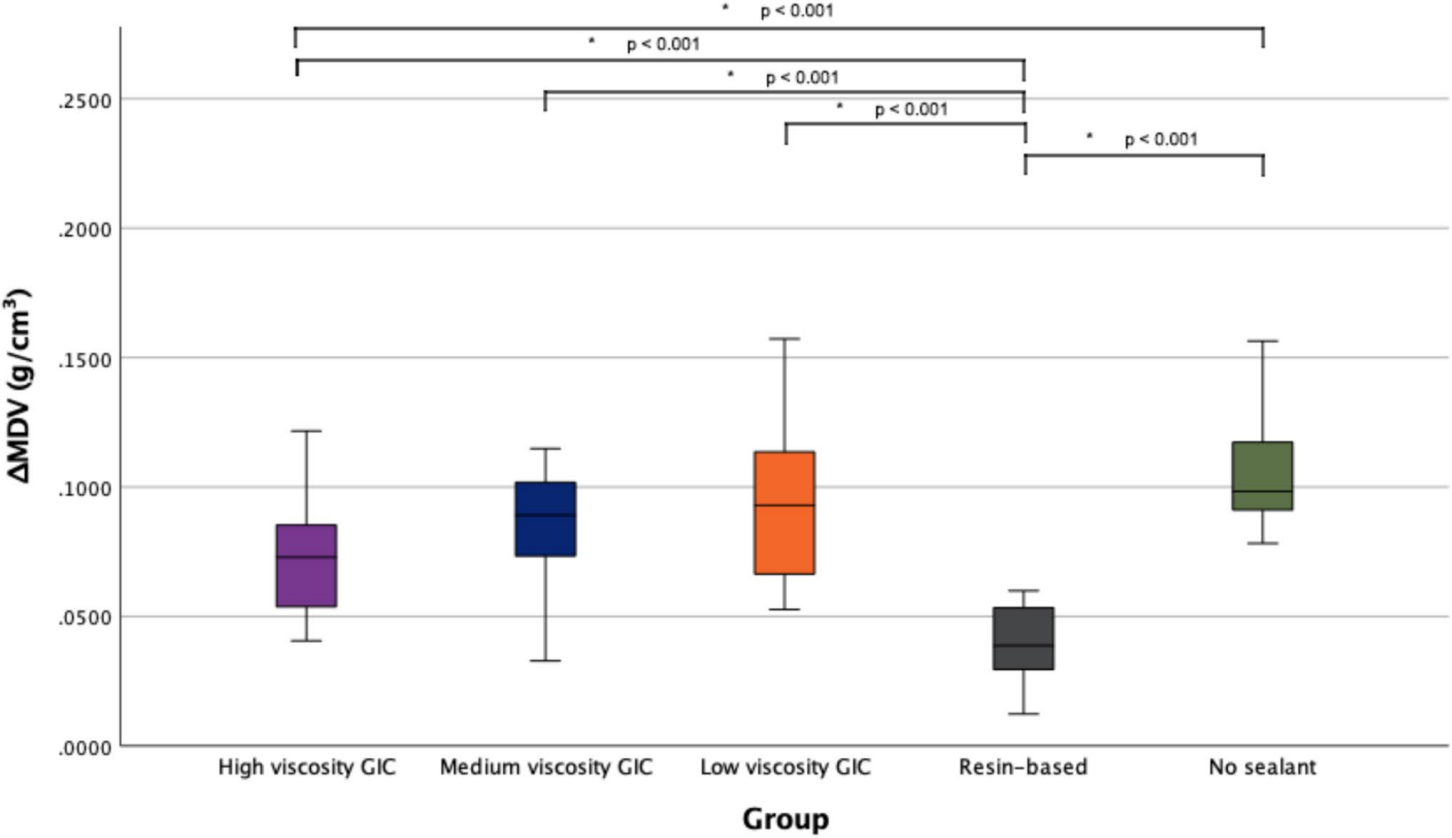
Mineral Density Changes (ΔMDV) of pH cycling test. The asterisk (*) indicates significant differences amongst the groups

Figure 5 shows the mineral density changes of pH cycling test between groups. The Kruskal–Wallis test revealed significant difference amongst the five groups (p < 0.001). Mann–Whitney U test with Bonferroni correction (α = 0.005) showed significant difference in pairwise comparisons. The RBS (Group 4) exhibited the lowest median ΔMDV (0.0387, IQR: 0.0252) which was significantly lower than high-, medium-, and low-viscosity GIS groups (Group 1,2,3, p < 0.001) and the group without sealants (Group 5, p < 0.001). No significant differences were observed between high-, medium-, and low-viscosity GIS groups, suggesting comparable performance in mineral retention (Table 3).

**Table 3:** Mineral Density Change (ΔMDV) of pH cycling amongst the groups

Group	Sealant type	Sealant brand (manufacturer)	Minimum ΔMDV (g/cm^3^)	Maximum ΔMDV (g/cm^3^)	Median ΔMDV (g/cm^3^)	IQR	Kruskal–Wallis *p *value
Group 1	High-viscosity glass-ionomer	Ketac™ Molar Easymix (3 M ESPE, MN, USA)	0.0406	0.1216	0.0728^b^	0.0359	*p* < 0.001
Group 2	Medium-viscosity glass-ionomer	GC Fuji VII (GC, Tokyo, Japan)	0.0329	0.1453	0.0891^bc^	0.029	
Group 3	Low-viscosity glass-ionomer	GC Fuji TRIAGE (GC, Tokyo, Japan)	0.0527	0.1572	0.0929^bc^	0.0282	
Group 4	Resin-based	Helioseal® (Ivoclar, Schaan, Liechtenstein)	0.0123	0.0600	0.0387^a^	0.0252	
Group 5	No sealant	Not applicable	0.0782	0.1564	0.0983^c^	0.0294	

Superscript letters indicate statistically homogeneous subsets based on Mann–Whitney *U* tests with Bonferroni correction (*p* ≤ 0.005). Groups sharing a letter are not significantly different.

Variables for multiple linear regression analysis: penetration rate (%), retention (partially intact, fully intact)

### Mineral density changes (ΔMDV) after bacterial test

**Fig. 6:**
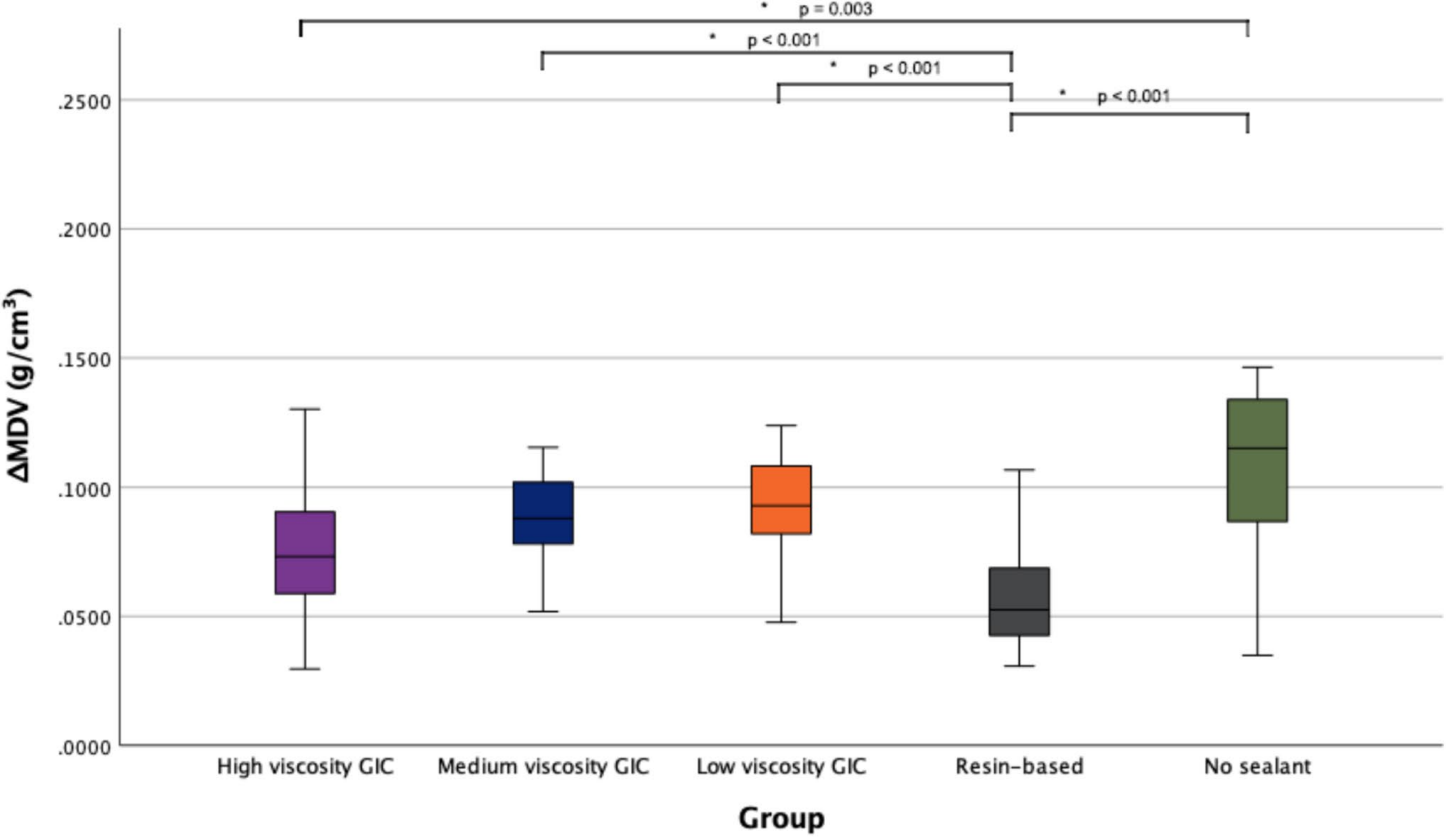
Mineral Density Changes (ΔMDV) of bacterial test. The asterisk (*) indicates significant differences amongst the groups.

Table 4 shows the minimum, maximum, median, and IQR of the ΔMDV from each group with the pairwise comparisons of ΔMDV using Mann–Whitney U tests. The Kruskal–Wallis test revealed significant difference amongst the five groups (p < 0.001). Mann–Whitney *U* test with Bonferroni correction (α = 0.005) showed significant difference in pairwise comparisons. Figure 6 shows the mineral density changes of bacterial test between groups. The RBS (Group 4) exhibited the lowest median ΔMDV (0.0526, IQR: 0.0349). This was significantly lower than the medium-viscosity (Group 2, *p* < 0.001) and low-viscosity glass-ionomer (Group 3, *p* < 0.001) sealant groups, as well as the group without sealant (Group 5, *p* < 0.001). Similarly, the high-viscosity GIS group (Group 1) had significantly lower median ΔMDV (0.0732, IQR 0.0336) when compared to the group without sealant (Group 5, p = 0.003). However, no significant difference was found between RBS (Group 4) and high-viscosity GIS (Group 1, p = 0.027), indicating comparable mineral retention performance between these two types of sealants.

**Table 4:** Mineral Density Change (ΔMDV) of bacterial test amongst the groups

Group	Sealant type	Sealant brand (manufacturer)	Minimum ΔMDV (g/cm^3^)	Maximum ΔMDV (g/cm^3^)	Median ΔMDV (g/cm^3^)	IQR	Kruskal–Wallis *p *value
Group 1	High-viscosity glass-ionomer	Ketac™ Molar Easymix (3 M ESPE, MN, USA)	0.0296	0.1302	0.0732^ab^	0.0336	*p* < 0.001
Group 2	Medium-viscosity glass-ionomer	GC Fuji VII (GC, Tokyo, Japan)	0.0519	0.1155	0.0879^bc^	0.025	
Group 3	Low-viscosity glass-ionomer	GC Fuji TRIAGE (GC, Tokyo, Japan)	0.0477	0.8654	0.0929^bc^	0.0282	
Group 4	Resin-based	Helioseal® (Ivoclar, Schaan, Liechtenstein)	0.0307	0.1096	0.0526^a^	0.0349	
Group 5	No sealant	Not applicable	0.0618	0.9967	0.1152^c^	0.0487	

Superscript letters indicate statistically homogeneous subsets based on Mann–Whitney *U* tests with Bonferroni correction (*p* ≤ 0.005). Groups sharing a letter are not significantly different.

Variables for multiple linear regression analysis: penetration rate (%), retention (partially intact, fully intact)

### Relationship between sealant retention, penetration ability, and mineral density changes under pH cycling and bacterial tests

The Mann–Whitney U tests showed no significant differences in penetration ability (p = 0.506), mineral density changes under pH cycling (p = 0.353), or bacterial test (p = 0.699) amongst groups categorised by sealant retention types. Spearman’s correlation analysis indicated no significant correlation between sealant penetration ability and ΔMDV under pH cycling (ρ = –0.148, p = 0.216) or bacterial test (ρ = –0.154, p = 0.197). However, a moderate and statistically significant positive correlation was found between mineral density loss under pH cycling and bacterial testing conditions (ρ = 0.463, *p* < 0.001), indicating that samples with greater demineralisation in one model tended to show similar behaviour in the other.

For the pH cycling model, the multiple linear regression was not statistically significant (p = 0.560, R^2^ = 0.054), with neither retention (p = 0.785) nor penetration (p = 0.304) showing significant effects. Similarly, in the bacterial test model, the regression was not significant (p = 0.910, R^2^ = 0.009), and both predictors remained non-significant (retention: p = 0.669; penetration: p = 0.968).

## Discussion

This study took into account both sealant material types and viscosities to evaluate their effects on sealant retention, fissure penetration, and caries inhibition ability. Based on the results, the first null hypothesis regarding sealant retention could not be rejected, as no significant differences were observed amongst the groups. However, the second and third null hypotheses related to fissure penetration and occlusal caries inhibition were rejected, given the statistically significant differences identified between specific groups.

Sealing pits and fissures is widely recognised as an effective caries-preventive measure, particularly for protecting occlusal surfaces of primary and permanent teeth in children and adolescents who are at heightened risk of caries development. Amongst the various sealant materials available, RBS and glass-ionomer cements (GICs) have been extensively studied due to their distinct properties. Resin-based sealants are valued for their superior retention and penetration ability, attributed to their hydrophobic nature and strong adhesion to enamel. Conversely, GICs offer unique advantages, including fluoride release and moisture tolerance, making them beneficial in cases where isolation is challenging. Despite numerous investigations comparing the long-term efficacy of these materials for caries prevention, there remains no consensus regarding their relative superiority (Kashbour et al. 2020; Lam et al. 2021). This ongoing debate underscores the importance of studies like the present one, which seeks to evaluate and compare the retention, penetration and caries inhibition properties of sealants with varying viscosities, contributing to evidence-based recommendations for clinical practise.

This study compared the retention and penetration ability of GIS with varying viscosities and RBS. The results revealed no significant difference in retention amongst the tested materials, which contrasts with findings from previous studies, suggesting that GIS generally exhibit lower retention rates compared to RBS (Lam et al. 2021).

The discrepancy may be attributed to the limitations of the current experimental setup, as clinical simulations were restricted to water storage and thermocycling without incorporating dynamic masticatory forces or the enzymatic activity of saliva. These factors are known to influence the longevity of fissure sealants in vivo (Haricharan et al. 2019; Hassan and Mohammed 2019; Jafer et al. 2022).

In terms of penetration ability, the RBS available in the market demonstrated the highest penetration rate, consistent with its low viscosity and superior flow properties, allowing deeper infiltration into the fissure system (Markovic et al. 2011). Conversely, the high-viscosity GIS exhibited significantly lower penetration ability when compared to both the RBS and the medium-viscosity GIS. This difference can be explained by the material's higher viscosity, which likely impeded its ability to flow into the intricate morphology of the fissures. However, it is noteworthy that all the sealant materials evaluated in this study achieved an overall penetration rate exceeding 90%, suggesting their effectiveness in adequately sealing fissures under the controlled experimental conditions, which aligns with findings from previous studies that also reported satisfactory sealing efficacy of both RBS and GIS (Garg et al. 2018).

In this study, pH cycling and *S. mutans* bacterial test were conducted to investigate the occlusal caries inhibition properties of sealants. The pH cycling model is widely used as it simulates the dynamic fluctuations of pH in the oral cavity, which occur due to the food intake and subsequent salivary buffering (Buzalaf et al. 2010). The demineralisation and remineralisation cycles can provide insights into the protective efficacy of fissure sealants under acidic challenges. This 21-day pH cycling protocol falls within the typical range of 3–35 days observed across various studies. Shorter durations, such as 7–10 days, may be sufficient for initial lesion formation or basic efficacy assessments. In contrast, longer durations, like 21–28 days, better simulate extended oral environmental stresses, such as those experienced by high-caries-risk patients with reduced saliva flow or frequent acid exposures (Fu et al. 2024).

Additionally, the *S. mutans* bacterial test is conducted to assess the cariogenic potential of oral biofilms, as it is a primary contributor to caries formation (Loesche 1986). By incorporating both pH cycling and bacterial tests, this study comprehensively evaluated the anti-demineralisation and anti-bacterial properties of sealants, mimicking real conditions in which both chemical and microbial factors contribute to caries development.

Thermocycling was conducted following the standardised testing protocol outlined in ISO 11405:2015 and previously established protocols, which is widely used to assess the durability of dental materials by replicating approximately one year of temperature fluctuations resulting from eating and drinking. Thermal stress effects and separate wear tests were highlighted in this method to isolate the effects of mastication, thereby ensuring clarity of the results without confounding factors (AlQahtani et al. 2022).

Resin-based fissure sealant consistently showed the lowest median ΔMDV in both pH cycling and bacterial tests, indicating its superior protection against mineral loss. In the pH cycling model, the RBS group exhibited significantly lower ΔMDV compared to high-, medium-, and low-viscosity GIS groups. Similarly, in the bacterial test, the RBS group performed superior resistance to enamel demineralisation, further confirming its anti-cariogenic ability. The strong protection of RBS may be attributed to several characteristics including higher penetration ability, better adhesion to enamel, and greater resistance to acid diffusion, which can help to reduce the enamel demineralisation under both chemical and microbial challenges (Naaman et al. 2017).

Conversely, the group without sealant demonstrated the highest ΔMDV in both tests, showing the greatest mineral density loss under acidic conditions and bacteria exposure. The significantly higher ΔMDV observed in group without sealant compared to high-viscosity GIS and RBS underscores the importance of sealant application in preventing enamel demineralisation, which were also stated in several other studies (Lam et al. 2021).

The GIS groups exhibited some caries protection effects, showing no significant differences amongst them in either test. This phenomenon suggests that despite differences in viscosity, these sealants provide comparable levels of anti-demineralisation capacity. Similar results were reported in the study by Cabral et al. (Cabral et al. 2018), which demonstrated comparable effectiveness of different types of GIS in caries prevention. However, their performances were consistently inferior to the RBS except when the high-viscosity GIS was compared to RBS under bacterial test (p = 0.027). The lower caries protection results of GIS contrasts with findings from several clinical trials. These trials reported no statistically significant difference in caries prevention between RBS and GIS, both of which showed favourable caries inhibition ability (Haricharan et al. 2022; Lam et al. 2021; Reić et al. 2022).

The findings of the current study suggested significant performance differences between GIS with different viscosities. However, in contrast to the findings of this study, evidence from clinical trials suggests that the caries prevention performance of GIS of different viscosities is comparable to that of resin-based sealants (RBS). Nonetheless, GIS, particularly those with higher viscosities, facilitate application using a finger press technique in compromised settings where moisture control is challenging (Mickenautsch & Yengopal 2016). Although RBS demonstrated superior caries inhibition under pH cycling and bacterial testing, clinicians should consider balancing its enhanced preventive effect with other handling properties and patient-specific factors.

The discrepancies between this study and previous clinical trials may be attributed to several methodological differences. Clinical trials are conducted in a real oral environment, where salivary buffering effect, dietary habit, fluoride exposure from water or toothpaste, and patient compliance influence caries progression, whereas these factors were absent in the controlled in vitro study. Additionally, the longer observational duration in clinical trials may enhance the fluoride-releasing effects of GIS, which can be more apparent over time. The acidic and bacterial conditions imposed in this study may have been harsher than those encountered in vivo, potentially exaggerating the demineralisation effects. Furthermore, clinical trials typically assess caries prevention using DMFT/dmft scoring, which evaluates caries at a macroscopic level, whereas this study employed micro-CT analysis, a more sensitive technique for detecting early stage mineral density changes.

Additionally, our study showed that neither sealant retention nor sealant penetration rates influenced the mineral density outcomes. The consistently high retention rates and penetration abilities (> 90%) may have limited statistical sensitivity to detect significant predictive relationships. In addition to sealant retention and penetration ability, other critical factors may potentially contribute to caries prevention, including fissure morphology, sealant composition, adhesive strength, marginal adaptation and integrity, wear resistance, and ions releasing functions (Garg et al. 2018; Ibrahim et al. 2021).

The findings of our study underscore the importance of material selection and sealant application in preventing occlusal caries, highlighting the superior performance of RBS compared to glass-ionomer alternatives. Future studies should focus on long-term, in vivo research to validate these findings under real oral conditions, considering factors, such as salivary interactions, dynamic masticatory force, wear resistance, and patient compliance. Whilst RBS may offer superior penetration ability due to their flow characteristics, GIS provide additional benefits, such as fluoride release and ease of application in moist environments, which make them particularly advantageous in specific patient populations, such as those with high caries risk or limited cooperation during application.

There are some limitations in the present study. This in vitro study was conducted under controlled laboratory conditions that do not fully replicate the complexity and the dynamic environment of the oral cavity. Variables, such as salivary flow, masticatory forces, and patient behaviour, were not simulated, which may limit the direct clinical applicability of the findings. Although fissure and sealant depths were recorded, natural anatomical variations amongst teeth may have influenced sealant penetration, introducing potential confounding. Blinding was not possible due to the distinctive appearance of different sealants under visual inspection and SEM assessment, which might induce potential outcome assessment bias. Mechanical loading was not combined in the thermocycling process; in addition, the long-term effects of material degradation and wear over time were not assessed. After attaining the results of these initial assessments, future research could consider combining thermocycling with mechanical loading and evaluating for a longer period of time to better simulate the masticatory forces experienced in the oral environment.

## Conclusion

Within the limitation of this in vitro study, the findings can be concluded as:

1. All tested fissure sealant, regardless of material type or viscosity, exhibited optimal penetration and retention abilities.
2. Resin-based fissure sealant demonstrated superior occlusal caries inhibition capacity.
3. GIS of different viscosities showed comparable caries inhibition capacities.

## Data Availability

The data generated from this study are available upon reasonable request to the corresponding author.
